# The Presence of Adipose Tissue in Aortic Valves Influences Inflammation and Extracellular Matrix Composition in Chronic Aortic Regurgitation

**DOI:** 10.3390/ijms26073128

**Published:** 2025-03-28

**Authors:** Alba Sádaba, Mattie Garaikoetxea, Carolina Tiraplegui, Susana San-Ildefonso-García, Miriam Goñi-Olóriz, Amaya Fernández-Celis, Ernesto Martín-Núñez, Paula Castillo, Virginia Álvarez, Rafael Sádaba, Eva Jover, Adela Navarro, Natalia López-Andrés

**Affiliations:** 1 Cardiovascular Translational Research, Navarrabiomed (Fundación Miguel Servet), Instituto de Investigación Sanitaria de Navarra (IdiSNA), Hospital Universitario de Navarra (HUN), Universidad Pública de Navarra (UPNA), 31008 Pamplona, Spain; alba.sadaba.cipriain@navarra.es (A.S.); mattie.garaikoetxea.zubillaga@navarra.es (M.G.); carolina.tiraplegui.garjon@navarra.es (C.T.); susana.sanildefonso.garcia@navarra.es (S.S.-I.-G.); miriam.goni.oloriz@navarra.es (M.G.-O.); amaya.fernandez.decelis@navarra.es (A.F.-C.); ernesto.martin.nunez@navarra.es (E.M.-N.); paulacristinacastillo@gmail.com (P.C.); virginia.alvarez.asiain@navarra.es (V.Á.); jr.sadaba.sagredo@navarra.es (R.S.); eva.jover.garcia@navarra.es (E.J.); 2 French-Clinical Research Infrastructure Network (F-CRIN) Cardiovascular and Renal Clinical Trialists (INI-CRCT), 54500 Nancy, France

**Keywords:** extracellular matrix, inflammation, adipocytes, valve interstitial cells

## Abstract

Adipose tissue is present in aortic valves (AVs). Valve interstitial cells (VICs) could differentiate into adipogenic lineages. We here characterize whether the presence of adipose tissue in the AV influences inflammation and extracellular matrix (ECM) composition in patients with aortic regurgitation (AR). A total of 144 AVs were analyzed by histological and molecular techniques. We performed discovery studies using Olink Proteomics^®^ technology in 40 AVs (N = 16 without and N = 24 with adipose tissue). In vitro, human white adipocytes (HWAs) or VICs were cultured with adipogenic media and co-cultured with control VICs. Of Avs, 67% presented white-like adipocytes within the spongiosa. Discovery studies revealed increased levels of inflammatory and ECM molecules in AVs containing adipocytes. Interestingly, the presence of adipocytes was associated with greater AV thickness, higher inflammation, and ECM remodeling, which was characterized by increased proinflammatory molecules, collagen, fibronectin, proteoglycans, and metalloproteinases. AV thickness positively correlated with markers of adipose tissue, inflammation, and ECM. In vitro, adipocyte-like VICs expressed higher levels of adipocyte markers, increased cytokines, fibronectin, decorin, and MMP-13. Analyses of supernatants from co-cultured control VICs with HWA or adipocyte-like VICs showed higher expression of inflammatory mediators, collagen type I, proteoglycans, and metalloproteinases. AVs presenting adipocytes were thicker and exhibited changes characterized by increased inflammation accompanied by aberrant expression of collagen, proteoglycans, and metalloproteinases. VICs could differentiate into adipogenic pathway, affect neighbor VICs, and contribute to inflammation, collagen and proteoglycan accumulation, as well as to metalloproteinases secretion. In summary, the presence of adipose tissue in AV could modify its composition, favoring inflammation and remodeling with an impact on AV thickness.

## 1. Introduction

Aortic regurgitation (AR) is characterized by a pathologic derangement of the aortic valve (AV) or loss of aortic root geometry [1]. Its prevalence in the United States is reported to be between 4.9% and 10% [2]. The pathophysiology underlying AR is poorly characterized and incompletely understood, thus limiting the development drug-based therapies alternative to the current surgical approach. The pathological processes include active AV remodeling and regulatory mechanisms. Valvular interstitial cells (VICs) exhibit a high degree of phenotypic plasticity and play a major role in the maintenance of the extracellular matrix (ECM) integrity [3]. The ECM presents a complex architecture, encompassing collagen, proteoglycans, and other molecules. Homeostasis of the AV’s ECM is regulated by a balanced secretion of matrix degradation enzymes, including matrix metalloproteinases (MMPs), A disintegrin-like and metalloproteases (ADAMTs), and their inhibitors (TIMPs) [4]. Of note, dysregulation of ECM remodeling could result in several pathological conditions, including inflammation. Proinflammatory cytokines play a critical role in maintaining the balance in ECM remodeling [5], mainly activating resident VICs [6]. Importantly, a study of proteomic mapping of 16 different heart regions described that the cardiac valves have the highest percentage of ECM-related protein expression as compared to other heart regions [7]. Depending on the stimuli and physical environment, VICs can undergo myofibroblastic, adipocytic, and osteogenic differentiation [8]. This phenotype plasticity affects their function and is associated with ECM remodeling [9]. Compared with myofibroblastic and osteogenic, the VIC-to-adipocyte transdifferentiation has been poorly studied [8].

The presence of adipose tissue (AT) within the AV has been shown in a few clinical case reports, although its role in AV pathophysiology remains unexplored [10,11]. AT has been found in both structurally normal and diseased AVs, mainly correlated with age [12]. Noteworthy, AT presence is usually concomitant with membranous fat necrosis, a distinctive degenerative process of mature adipocytes. Thus, AVs with adipocytes exhibit greater mean AV area as compared to those without adipocytes [10]. Moreover, the presence of AT was greater amongst AVs with moderate aortic stenosis, whereas the presence of degenerative adipocytes was higher in moderate AR [10], suggesting their involvement in the pathology of the AV by different means.

The aim of the present study was to characterize the presence of AT in AVs from AR patients and its association with inflammation and the ECM changes found in AR. Moreover, we aimed to explore whether the VIC may replenish the adipocyte counts within the AV towards adipogenic transdifferentiation potentially impacting the inflammatory profile and the ECM composition of the AV.

## 2. Results

### 2.1. Clinical and Histological Parameters in AR Patients Presenting Adipocytes

**Figure 1:**
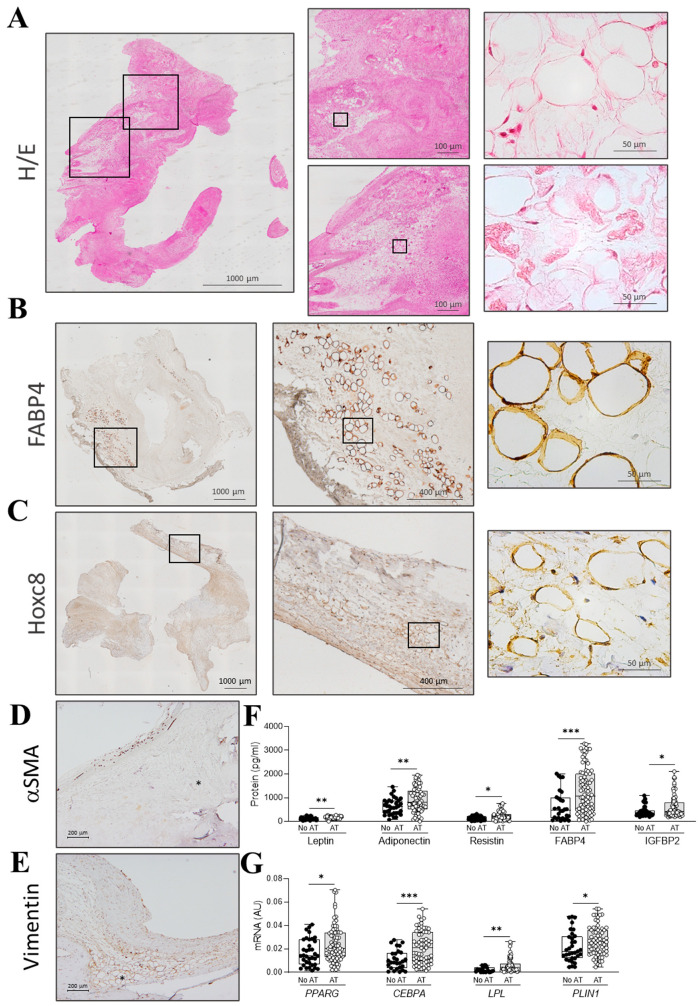
Characterization of AT presence in AVs from AR patients. Panoramic and augmented images of AVs from AR patients stained with hematoxylin–eosin (**A**), or immunostained for FABP4 (**B**), Hoxc8 (**C**), α-SMA (**D**), and vimentin (**E**). Box plots showing all individual values for the expression of leptin, adiponectin, resistin, FABP4, and IGFBP2 at the protein level (**F**) and *PPARG*, *CEBPA*, *LPL*, and *PLIN1* at the mRNA levels (**G**) measured in 144 AVs (N = 96 with AT, N = 48 without AT). Asterisk on the microphotographs indicates the presence of adipocytes. Black dot blots represent AV without AT and white dot blots represent AV with AT. * *p* < 0.05, ** *p* < 0.01, *** *p* < 0.001.

Histological screening was performed in AVs. The presence of AT was documented in the spongiosa layer of 67% of the AVs in hematoxylin–eosin-stained sections (Figure 1A) and confirmed in FABP4 (Figure 1B) and Hoxc8 (Figure 1C) immunostained sections. Moreover, in order to show the spatial association among adipocytes and VICs, α-SMA, a marker of activated VICs (VICs with a myofibroblastic phenotype), immunohistochemistry was performed in AVs encompassing adipocytes. As shown in the representative microphotograph, activated VICs were not found close to the adipocytes (Figure 1D). Complementarily, vimentin immunostaining showed positivity in VIC- and adipocyte-occupied regions (Figure 1E).

AVs with AT (N = 96) exhibited higher levels of classical adipose tissue markers both at the protein and mRNA levels: leptin, adiponectin, resistin, FABP4, and IGFBP2 (Figure 1F) and *PPARG*, *LPL*, and *PLIN1* (Figure 1G).

No different clinical characteristics were found in the presence of AT (Table 1). Of importance, the presence of AT was similar between sexes (Table 1). Echocardiographic and computed tomography parameters were those estimated in patients with severe AR. AV thickness was higher in AVs with AT than in those without AT. Moreover, the presence of adipocytes was positively correlated with the AV thickness. Other parameters, such as ejection fraction, were similar regardless of the presence of AT (Table 1).

### 2.2. Targeted Proteomics Reveals Changes in ECM Molecules in AVs with Adipocytes

**Figure 2:**
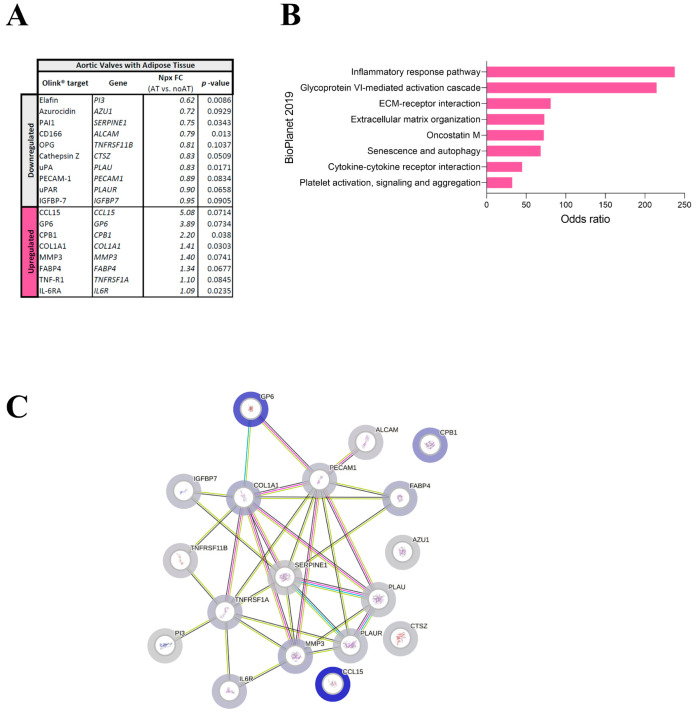
Identification of targets differentially expressed in AVs with and without AT. AVs with AT showed different over-expressed targets (**A**) and molecular pathways (**B**). Protein–protein interaction network of the differentially expressed targets in AVs without and with AT, protein blue halo color showing proportionally upregulated proteins (**C**).

Multivariate analysis of the Olink Proteomics^®^ panel data showed that 18 targets were differentially expressed in AVs with AT as compared with AVs without AT (8 up-regulated and 10 down-regulated) (Figure 2A). Further enrichment analysis considering adipocyte presence in AVs showed that in AVs with AT, there was a higher presence of inflammatory and ECM pathways, while in AVs without AT, the plasminogen activator system pathway was enhanced (Figure 2B). Accordingly, protein pathway analysis showed positive interactions between most of the proteins found differentially regulated in the Olink panel. Remarkably, in AV with AT, proteins involved in ECM and inflammation exhibited positive interactions (Figure 2C).

### 2.3. Inflammation and ECM Composition in AVs with Adipocytes

**Figure 3:**
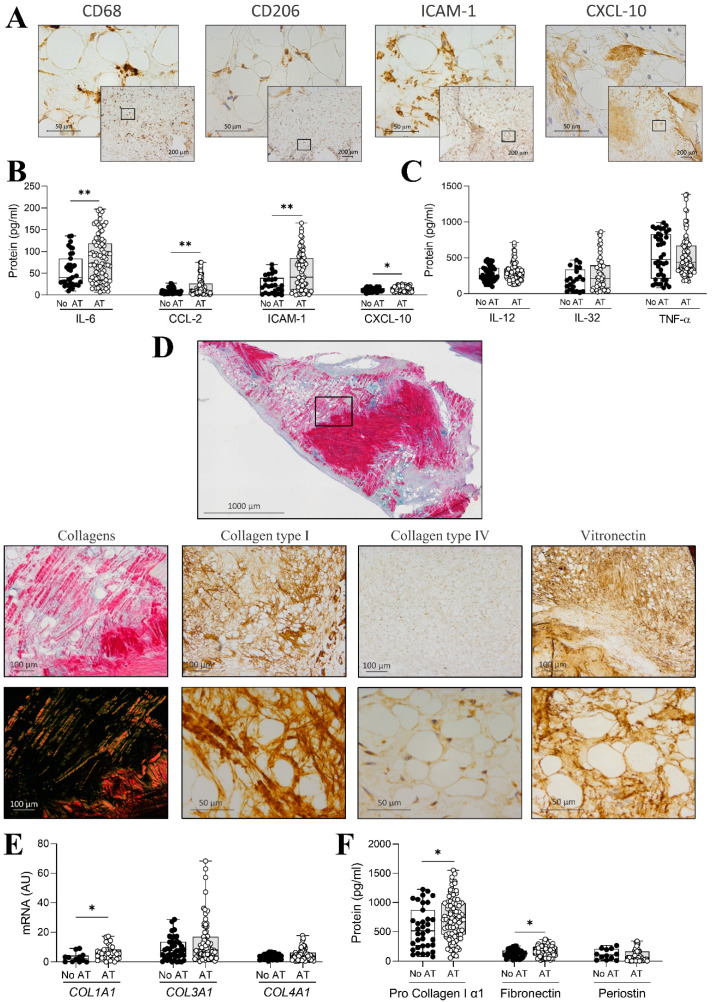
Inflammation and ECM composition in AVs with and without AT. Representative microphotographs of AV sections from patients with AR with adipocytes immunostained for CD68, CD206, ICAM-1, and CXCL10 (**A**). Box plots showing all individual values for the expression of IL-6, CCL-2, ICAM-1, and CXCL10 (**B**) and IL-12, IL-32, and TNF-α (**C**) in whole AVs harvested from AR patients. Representative microphotographs of AV sections from patients with AT stained with Sirius red-alcian blue under bright or polarized light and immunostained for collagen type I, collagen type IV, and vitronectin (**D**). Box plots showing all individual values for the expression of *COL1A1*, *COL3A1*, and *COL4A1* at the mRNA level in whole AVs harvested from AR patients (**E**). Box plots showing all individual values for the expression of collagen type I, fibronectin, and periostin at the protein level (**F**) in whole AVs harvested from AR patients. N = 144 AVs (N = 96 with AT, N = 48 without AT). Black dot blots represent AV without AT and white dot blots represent AV with AT. * *p* < 0.05, ** *p* < 0.01.

Considering the results of the discovery approach, inflammatory pathways were further explored in AVs with and without AT. Immunohistochemical analyses of AV sections revealed the presence of CD68- and CD206-positive cells surrounding adipocytes (Figure 3A). Accordingly, adipocytic ECM exhibited positive immunostaining for the inflammatory ICAM-1 and CXCL-10 molecules (Figure 3A). To characterize the influence of AT on inflammation, inflammatory markers were measured in AV protein homogenates of the whole AR cohort by ELISA (Figure 3B,C). AVs with AT showed significantly higher expression of IL-6, CCL-2, ICAM-1, and CXCL10 (Figure 3B), while no differences were found for IL-12, IL-32, and TNF-α (Figure 3C).

Following the pathways suggested by the enrichment analysis, ECM composition was additionally characterized in AVs with and without AT. Panoramic microphotographs of AVs with AT double stained with Sirius red and alcian blue showed collagen accumulation in areas surrounding AT (Figure 3D). Supplementary analysis under polarized light revealed thick collagen fibers in AVs with AT, which were confirmed by collagen type I immunostaining (Figure 3D). Moreover, AT-occupied areas were also positive for collagen type IV and vitronectin, an ECM protein which binds with major ECM receptors, integrins, and other ECM components, such as collagen and proteoglycans (Figure 3D). Complementarily, quantification of collagens was performed in AVs with and without AT. AVs with AT exhibited higher levels of mRNA of *COL1A1* (*p* = 0.033), whereas *COL3A1* (*p* = 0.1792) and *COL4A1* (*p* = 0.1220) expressions did not vary between AVs with and without AT (Figure 3E). Furthermore, collagen type I protein expression was higher (*p* = 0.0301) in AVs with AT (Figure 3F). In order to expand these results, other ECM components were quantified in tissue homogenates from AV without and with AT. AVs with AT expressed higher levels of fibronectin (*p* = 0.0320) and similar levels of periostin/*POSTN* (Figure 3F).

**Figure 4:**
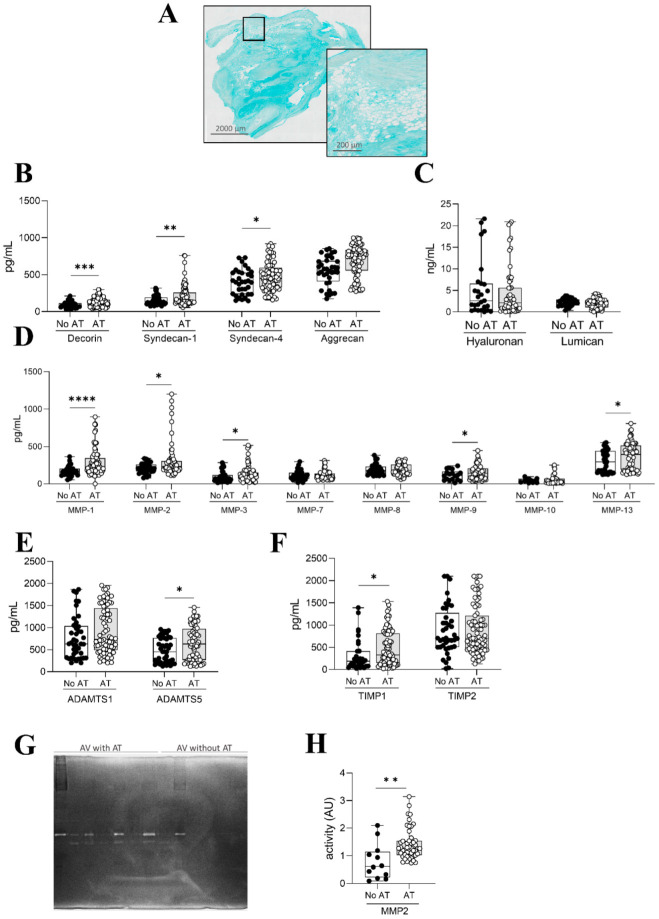
Proteoglycans expression and ECM remodeling in AVs with and without AT. Alcian blue staining showing proteoglycans content in AV with AT (**A**). Decorin syndecan-1, syndecan-4, and aggrecan (**B**) and hyaluronan and lumican (**C**) expression at the protein level in whole AVs. Box plots showing all individual values for the expression of MMPs-1, -2, -3, -7, -8, -9, -10, and 13 at the protein level in whole AVs harvested from AR patients (**D**). Box plots showing all individual values for the expression of ADAMTs1 and ADAMTs5 at the protein level (**E**) as well as for TIMP1 and TIMP2 at the protein level (**F**) in whole AVs harvested from AR patients. Representative gelatin zymograms for MMP-2 (**G**). MMP-2 activity quantification in whole AVs (**H**). N = 144 AVs (N = 96 with AT, N = 48 without AT). Black dot blots represent AV without AT and white dot blots represent AV with AT. * *p* < 0.05, ** *p* < 0.01, ***, *p* < 0.001, **** *p* < 0.0001.

As interactions between collagens and proteoglycans define the structure and function of ECM, proteoglycan distribution and concentration were explored in AVs with and without AT. Panoramic microphotographs stained with alcian blue showed proteoglycans distribution in AVs with AT (Figure 4A). Then, the quantification of proteoglycans revealed increased levels of decorin (*p* = 0.0003), syndecan-1 (*p* = 0.0262), syndecan-4 (*p* = 0.0134), and aggrecan (*p* = 0.0447) in AVs with AT (Figure 4B). Levels of lumican and hyaluronan were similar in AVs with and without AT (Figure 4C).

Homeostasis of the AV ECM is regulated by the deposition of structural ECM components and a balanced secretion of matrix degradation enzymes (MMPs, ADAMTs) and their inhibitors (TIMPs). Therefore, we next examined the expression of MMPs, ADAMTs and TIMPs in AVs with and without AT. The expression of the degrading enzymes MMP-1 (*p* < 0.0001), -2 (*p* = 0.0297), -3 (*p* = 0.0109), -9 (*p* = 0.0281), and -13 (*p* = 0.0392) as well as ADAMTs5 (*p* = 0.0409) was enhanced in AVs containing AT as compared to AVs without AT (Figure 4D–F). However, MMP-7, -8, -10, and ADAMTs1 levels were similar in both types of AVs (Figure 4D,E). The MMP inhibitor TIMP1 was increased (*p* = 0.0167) in AVs with AT, whereas TIMP2 levels were similar in AVs with and without AT (Figure 4F). Accordingly, MMP-2 zymography revealed higher MMP-2 activity in AVs with AT (Figure 4G,H).

### 2.4. Correlations Between AV Thickness with Inflammation and ECM Components in AR Patients

**Figure 5:**
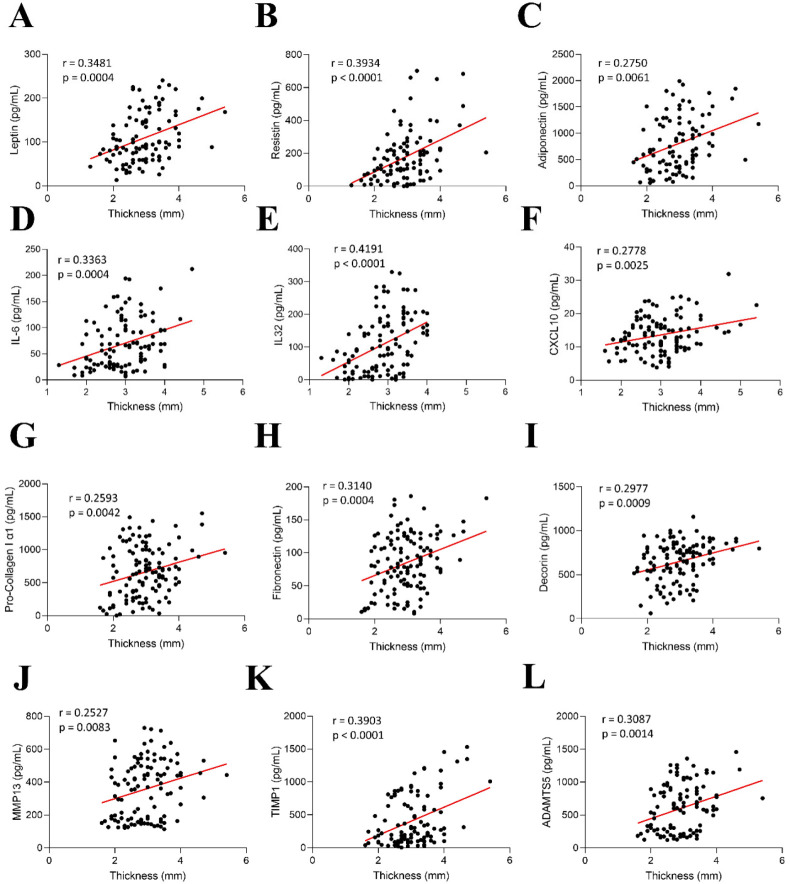
Aortic valve thickness correlations with tissue damage markers. Association of AV thickness with leptin (**A**), resistin (**B**), adiponectin (**C**), IL-6 (**D**), IL-32 (**E**), CXCL-10 (**F**), procollagen type I (**G**), fibronectin (**H**), decorin (**I**), MMP-13 (**J**), TIMP1 (**K**), and ADAMTs5 (**L**).

Further correlation analyses showed correlations between AV thickness and the AT markers leptin (r = 0.3481, *p* = 0.0004), resistin (r = 0.3934, *p* < 0.0001), and adiponectin (r = 0.2750, *p* = 0.0061) (Figure 5A–C). Regarding inflammation, positive correlations were found between AV thickness and IL-6 (r = 0.3363, *p* = 0.0004), IL-32 (r = 0.4149, *p* < 0.0001), and CXCL-10 (r = 0.2778, *p* = 0.0025) (Figure 5D–F). In addition, AV thickness was positively correlated with the ECM molecules collagen type I (r = 0.2593, *p* = 0.0042), fibronectin (r = 0.3140, *p* = 0.0004), and the proteoglycan decorin (r = 0.2977, *p* = 0.0009) (Figure 5G–I). Noteworthy, AV thickness also correlated with the ECM degradation proteins MMP-13 (r = 0.2527, *p* = 0.0083), TIMP1 (r = 0.3903, *p* < 0.0001), and ADAMTs5 (r = 0.3087, *p* = 0.0014) (Figure 5J–L).

### 2.5. Characterization of Adipogenic Differentiation of VICs

**Figure 6:**
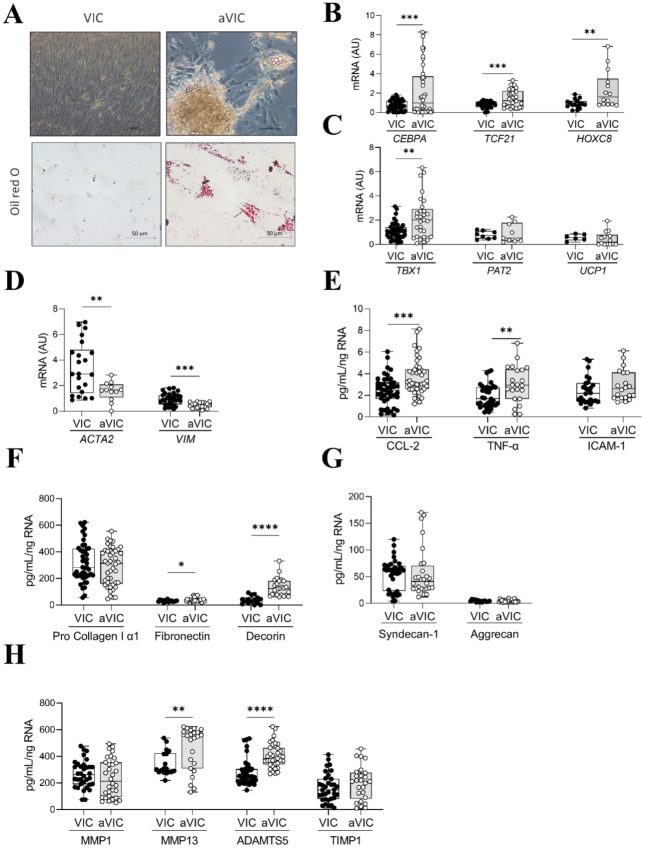
Analysis of the differentiation capacity in VICs isolated from AR patients. Microscopic visualization of lipid accumulation in bright field and in Oil Red O stained VICs isolated from AR patients and cultured for 28 days in standard growth medium (control) or adipogenic medium, as indicated (**A**). Boxplots of mRNA levels from control and adipogenic VICs of *CEBPA*, *TCF21*, *HOXC8* (**B**); *TBX1*, *PAT2*, *UCP1* (**C**); and *ACTA2*, *VIM* (**D**). Boxplots of secreted CCL-2, TNF-α, and ICAM-1 (**E**). Boxplots of secreted pro collagen I α1, fibronectin, decorin (**F**), syndecan-1, and aggrecan (**G**) from control and adipogenic VICs. Boxplots of secreted MMP-1, MMP-13, ADAMTs5, and TIM1 (**H**) from control and adipogenic VICs. Each dot represents a technic cell culture replicate. VIC, control VICs; aVIC, adipocyte-like VICs. VICs. * *p* < 0.05, ** *p* < 0.01, *** *p* < 0.001, **** *p* < 0.0001. N = 5 men-derived VICs.

In order to evaluate VIC differentiation capacity, VICs isolated from AR patients were cultured in adipogenic media for 28 days. Adipocyte differentiation was observed in VICs cultured in adipogenic media at day 28, as evidenced by the presence of lipid droplets in oil red O staining (Figure 6A). Differentiated VICs exhibited characteristics of white adipocytes, presenting increased expression of *CEBPA* (*p* = 0.0003), *TCF21* (*p* = 0.0008), and *HOXC8* (*p* = 0.0066) (Figure 6B). The beige adipocyte marker *TBX1* was also increased after 28 days of culture in adipogenic media, whereas *PAT2* expression was low and similar between control and adipogenic VICs (Figure 6C). The brown adipose tissue marker *UCP1* was poorly expressed by VICs and adipogenic VICs (Figure 6C). In accordance, VIC markers *ACTA2* and *VIM* were reduced (*p* = 0.0047 and *p* = 0.0004, respectively) in adipogenic VICs (Figure 6D). Regarding inflammation, adipogenic VICs secreted higher levels of CCL-2 and TNF-α than control VICs (Figure 6E). ICAM-1 secretion tended to be higher in adipogenic VICs although it did not reach statistical significance (Figure 6E). Concerning ECM components, VICs and adipogenic VICs secreted similar levels of collagen type I, aggrecan, and syndecan-1, although adipogenic VICs presented higher secretion of fibronectin (*p* = 0.0369) and decorin (*p* < 0.0001) (Figure 6F,G). The analysis of the enzymes involved in ECM degradation revealed that adipogenic VICs secreted higher levels of MMP-13 (*p* = 0.0079) and ADAMTS5 (*p* < 0.0001) with no major changes in MMP-1 or TIMP1 as compared to controls (Figure 6H).

### 2.6. Co-Culture of Adipogenic VICs with Control VICs

**Figure 7:**
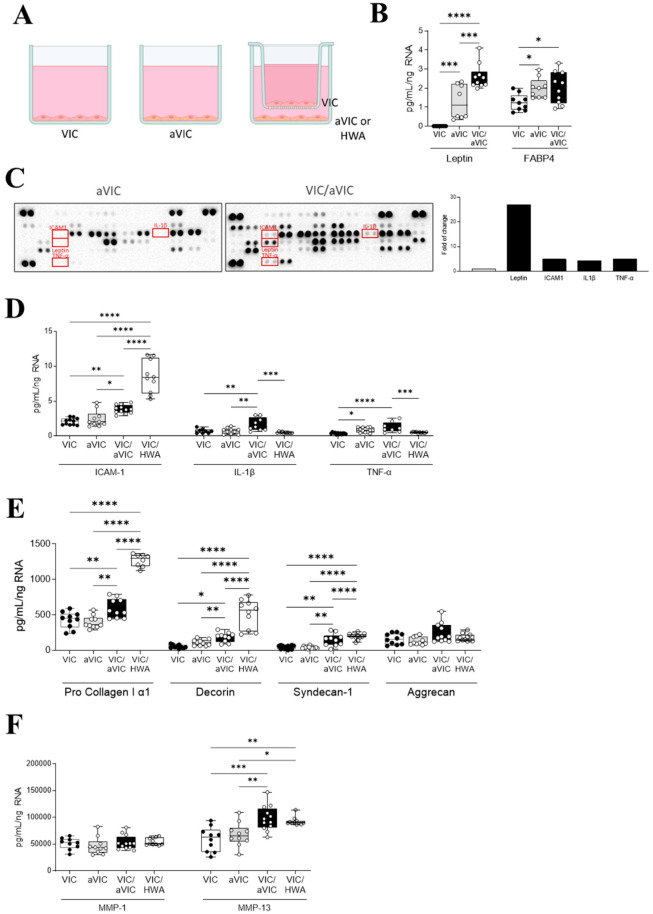
Co-culture of control VICs with adipogenic VICs isolated from AR patients or HWA. Experimental design and experimental groups (VIC, a VIC, co-culture VIC with aVIC or HWA) analyzed in this Figure (**A**). Boxplots showing all individual values for secreted leptin and FABP4 (**B**). Proteome profiler array dot blots from adipogenic and co-cultured control and adipogenic VICs derived from regurgitant AVs (N = 3 donors pooled per condition). Selected blots are red-squared within the dot blot membranes. Densitometric analyses were plotted upon normalization by the reference spots (internal controls) included within each dot blot membrane (**C**). Boxplots showing all individual values for secreted ICAM-1, IL-1β, and TNF-α (**D**); Pro Collagen I α1, decorin, syndecan-1, aggrecan (**E**); MMP-1 and MMP-13 (**F**). Secreted concentrations are normalized by RNA concentration of each well (co-cultures include RNA concentration of the sum of the well and the transwell). N = 3 men-derived VICs (donors). * *p* < 0.05, ** *p* < 0.01, *** *p* < 0.001, **** *p* < 0.0001.

In order to study whether factors secreted by adipocyte-differentiated VICs could influence resident VICs, we performed indirect co-culture experiments of control VICs with adipogenic VICs. Complementarily, human white adipocytes (HWA) were also co-cultured with primary VICs, with the objective to compare the effect with aVICs. Primary cultured VICs were plated on the bottom chambers and differentiated for 21 days into adipocyte-like VICs. Non-differentiated VICs from the same donor cultured in control media were separately plated on permeable polycarbonate membrane inserts on day 0 and cultured for 24 h to allow them to attach. After the 24 h of VIC anchoring, the seeded inserts (upper chambers) were moved together with the adipocyte-like VICs (bottom chambers) or the HWA, as represented in Figure 7A. Co-cultures were maintained for 7 days. The study of the adipocyte markers leptin and FABP4 were increased in adipogenic VICs as compared to VICs cultured in control media, which was exacerbated in co-cultured VICs with adipogenic VICs (Figure 7B).

A proteome profiler array was made to compare the secretion of inflammatory markers in monocultured control, in adipocytic VICs, and in co-cultures (Figure 7C). The following molecules were identified up-regulated in VIC/adipocyte-like VIC co-cultures as compared to monocultured VICs: leptin (270%), ICAM-1 (492%), IL1β (434%), and TNF-α (505%) (Figure 7C). Further validation analyses were performed by ELISA, normalizing all the readouts to the RNA concentrations in all the analyzed experimental conditions to avoid biased results arising from different cell counts.

VICs exposed to adipocytic VIC and HWA co-culture also secreted higher levels of ICAM-1 as compared with VICs cultured in control or adipogenic media, highlighting the greater increase in the co-culture with HWA conditions. Adipocyte-differentiated VICs showed higher levels of TNF-α. VICs exposed to adipocytic VIC also secreted higher levels of TNF-α and IL-1β compared to control VICs, although TNF-α levels were already increased in adipogenic VICs. The co-culture with HWA, in contrast, did not increase the secretion of these inflammatory molecules compared to control VICs (Figure 7D).

Collagen type I was increased in both co-culture conditions, emphasizing the higher levels of the VIC/HWA co-culture. The ECM-related proteoglycans decorin and syndecan-1 followed the same trend as the pro collagen I α1. Thus, the secretion of both decorin and syndecan-1 was enhanced in the co-culture conditions compared to control VIC and adipocytic VICs, being higher in the co-culture with HWA (Figure 7E). However, aggrecan proteoglycan levels did not vary between all the assayed conditions (Figure 7E).

The ability of adipocytic VICs to increase ECM markers was evidenced by quantifying the fibrosis markers, showing an increase of the secretion of collagen type I, decorin, syndecan-1, and aggrecan as compared with VICs cultured in control or adipogenic media (Figure 7C). MMP-1 levels remained unchanged in both co-cultured conditions compared with both control and adipogenic VICs. However, primary VICs exposed to adipocytic VIC and HWA co-culture for 7 days showed an increase in MMP-13 (Figure 7F).

## 3. Discussion

Our results showed that the presence of AT in the AV was associated with higher inflammation, ECM synthesis, and remodeling, contributing to AV thickening. Fresh VICs isolated from patients exhibited a potential of differentiation into adipocytes. Of note, adipogenic VICs predominantly expressed white fat markers, in line with the enhanced release of pro-inflammatory and ECM molecules relevant to the AV remodeling observed in AR. Moreover, adipocyte-like VICs directly or indirectly influenced neighboring VICs by producing soluble factors that increased inflammatory recruitment and ECM remodeling in a vicious circle. These findings indicate that the ability to modulate VICs’ phenotype may play a key role in AV pathophysiology.

The prevalence of AR is underestimated [1], and the cellular and molecular mechanisms orchestrating this phenomenon are unknown. In our study, the presence of adipocytes has been documented in 67% of the AVs obtained from AR patients, suggesting adipogenesis to be a relevant mechanism to the disease. Interestingly, adipocytes’ presence was not related with clinical characteristics of the AR patients, including their metabolic profile. This finding is well in line with the data showing that body mass index was similar among patients regardless of the presence of adipocytes in their cardiac valves [10]. Moreover, AT seems to change the microenvironment of the AV, turning it into one that is rather inflammatory and fibrotic. The replacement of valvular-to-adipose tissue has been indeed demonstrated by the presence of degenerated adipocytes in association with increasing functional regurgitation [10]. Now, we also demonstrated that such adipose tissue was mainly white AT, including the expression of leptin, adiponectin, and the master ‘adipose’ transcription factor *PPARG*, along with a weakened expression of brown and beige fat markers. Although the potential for AT to impact cardiovascular physiology has been studied in epicardial, pericardial, and perivascular localizations [13], resulting in enhanced inflammatory and fibrotic cues [14], the role of AT in the AV is largely unexplored. Accordingly, the presence of AT in AVs was associated with an increased inflammation and ECM turnover. It is well recognized that the composition of the ECM influences inflammatory cell activation [15]. Thus, ECM components, such as fibronectin and proteoglycans, can bind to cytokines, creating a local pro-inflammatory milieu. Of importance, we described adipocytes as a new source of inflammatory and ECM molecule in the AV. Therefore, the presence of adipocytes could contribute to inflammatory cell recruitment, modifying the composition, organization, and stability of the ECM and contributing to AV thickening.

Towards a high phenotype plasticity, the VIC contributes to the homeostasis and pathology of the AV. A growing number of publications reveals the VIC’s ability to undergo multiple phenotypes that might be pathologically relevant to different stages and forms of AV disease [16]. Most of the cellular studies have been focused on VIC-to-osteoblast differentiation [17]. However, it is known that AR is a non-calcific understudied pathology with surgical valve replacement as the only currently accepted therapeutic choice. Our study showed that VICs from AR patients could differentiate into functional adipocytes, exhibiting lipid droplet accumulation along with the expression of adipogenic markers, such as PPAR-gamma and adipokines, indicating that they have great potential for adipogenic differentiation [18]. In line with our findings, VICs isolated from healthy valves exhibit higher lipid accumulation compared to cells isolated from calcified valves when they are cultured in adipogenic media [19]. Previous publications proved the contribution of circulating progenitor cells to the pathogenesis of the naïve and bioprosthetic AV. Along with other multiple mesenchymal lineages, the adipogenic pathway is an independent characteristic of mesenchymal cell multipotency. We cannot rule out whether valvular circulating progenitor cells [20] might be recruited into the AV and act as an adipocyte reservoir relevant to the pathogenesis of AR. Nevertheless, our in vitro co-culture studies combining adipocyte-like VICs or HWA with undifferentiated control VICs may suggest that the presence of valvular adipocytes, of whatever cell source, can exert a recruiting pro-adipogenic effect on the neighboring VICs, in turn shown to be highly prone to undergo adipogenic differentiation. Thus, the higher expression of adipogenic markers in VICs from AR valves could suggest that they have higher potential for adipogenic differentiation.

Our results suggest that adipogenic VICs could change the microenvironment in the AV, affecting resident neighbor VICs and promoting inflammation and ECM remodeling. For instance, adipogenic VICs as well as fully differentiated HWA influenced the secretory profile of resident VICs, increasing the production of cytokines, collagen, proteoglycans, as well as the main ECM degrading enzymes. Consequently, thick collagen type I and vitronectin were located surrounding AT in the AV. It is interesting to point out the unknown role of vitronectin in heart valve diseases. However, our findings are consistent with previously reported interpretations showing that vitronectin brings the collagen fibers into close contact with each other increasing their thickness [21]. In line with these results, fibronectin, a glycoprotein known to be required for collagen fibril assembly [22], was also secreted by adipogenic VICs and enhanced in AVs with AT. Decorin as well as syndecans, known players in the assembly of collagen and fibronectin fibrils and in collagen crosslinking [5,23], were similarly secreted by adipogenic VICs and increased in AVs with AT.

Owing to their ability to cleave ECM components, the MMP and ADAMT families have been extensively investigated [24]. Remodeling of the AV ECM by proteases is crucial to establish the mechanical properties of the tissues, and facilitates infiltration of inflammatory cells [25]. In AVs with AT, there was an increase in MMPs-1, -2, -3, -9, and -13 as well as in TIMP1 expressions. Adipogenic VICs could contribute to this imbalance, and their presence could be associated with abnormal inflammation and ECM remodeling, including disorganized collagen and proteoglycans. Of note, AT presents chronic low-grade inflammation which may stimulate the VICs to express MMPs, thus actively regulating ECM remodeling [26]. Moreover, an increase in MMPs has been reported in myxomatous valves, rich in proteoglycans [27,28]. Additionally, AVs with AT exhibited higher levels of ADAMTs5. Accordingly, the proteolytic activity of ADAMTs5 is essential in regulating the levels of proteoglycans and disturbances in their levels could affect the disease process [29]. It is known that changes in ECM are critical in AR development [9,30], and a growing number of publications on mechanobiology certainly prove that changes in the strains of VICs can affect their function [31]. Taken together, our results suggest that the presence of AT could influence ECM remodeling by modifying the sophisticated AV architectural network.

## 4. Material and Methods

### 4.1. Patient Population

This prospective, observational study included a total of 144 consecutive patients with chronic severe symptomatic non-rheumatic AR diagnosed by echocardiography, according to clinical practice guidelines [32], who were referred to our center for AV replacement from 2014 to 2023. The main criteria for AV replacement were based on symptoms (NYHA > II, 55%) or left ventricular impairment according to the latest clinical practice guidelines. Patients with moderate or severe concomitant aortic valvular disease, previous or current infective endocarditis, inflammatory diseases, malignant tumor, Marfan disease, acute aortic syndromes, rheumatic disease, and advanced renal failure were excluded.

Informed consent was obtained from each patient, and the study protocol conforms to the ethical guidelines of the 1975 Declaration of Helsinki, as reflected in a prior approval by the institution’s human research committee (project 26/2013).

### 4.2. Echocardiographic and Multi-Detector Computed Tomography Measurements

Board-certified cardiologists with extensive expertise in echocardiography performed AR assessment, using high-end scanners (IE33 xMATRIX model Ultrasound Machine, Phillips, Dublin, Ireland). All patients were evaluated by transthoracic echocardiography according to the European Association of Cardiovascular Imaging [33].

Aorta parameters were also measured in 64 patients by multi-detector computed tomography (MDCT) using a using a 64 detector CT scanner (Phillips Brilliance 64, Brownsville, TX, USA).

AV thickness was defined as a focal area of increased echogenicity and thickening of the AV leaflets without restriction of leaflet motion [34]. Measurements were made by two separate observers who were blinded to each other’s measurements. Individual measurements were made three times from optimal still frames of cine-loops. Measurements were made in millimeters to an accuracy of one decimal. The thickness of the aortic valves tends to vary according to race and age; however, in adults, the normal thickness of the aortic valve typically ranges from 1.5 to 3 mm in transthoracic echocardiography [35]. In our study, we considered increased valve thickness when it exceeded 3 mm.

### 4.3. Histology and Immunohistochemistry

AV samples were fixed in 4% formaldehyde solution and then embedded in paraffin to generate 5-µm sections. Both hydration and dehydration procedures were conducted before and after staining. Automated Carazzi hematoxylin and alcoholic eosin staining (PanReac/Bio-Optica, Milano, Italy) were applied for 3 min and 5 min, respectively, utilizing a Leica Autostainer XL machine (Leica, Wetzlar, Germany). Alcian blue staining was performed by immersing samples in alcian blue (Sigma-Merck Life Science S.L.U., Madrid, Spain) for 20 min. Double staining with alcian blue and Sirius red followed the same procedure, adding a 30-min immersion in Sirius red solution (1% in picric acid, Sigma-Merck Life Science S.L.U., Madrid, Spain) after the alcian blue staining step. Light polarization was performed in these double-stained samples.

Immunohistochemistry procedures involved filling immunostaining solutions into the bottle-Bond Open Container (Leica) and logging them onto the Leica Biosystem program. The process included automated fixation, washing with bond wash solution, blocking using a common immunohistochemistry blocker, and incubation with antibodies.

Subsequently, poly-HRP-IgG incubation served as the secondary antibody. Immunopositive reactions were detected using DAB (3,3′-Diaminobenzidine). To conclude, panoramic views of some AV slides were achieved by compiling images captured at 5× magnification using Cytation 5 (BioTek, Winooski, VT, USA), with Gen5 Image+ software version 2.0.

### 4.4. AV Processing

The AV tissue was fragmented in 3 pieces. One fragment was frozen with liquid nitrogen and pulverized mechanically to create a homogenate, which was subsequently used for protein extraction with cOmplete™ Lysis-M EDTA-free buffer (Roche, Merck, Darmstadt, Germany) and extraction with guanidinium thiocyanate (PRImeZOL, Canvax, Córdoba, Spain). Another fragment was fixed in 4% formaldehyde for histologic analysis while the last one was used for VIC extraction as a laboratory routine [36]. To avoid variability, all AV samples were cut at the same level of the leaflet to perform histological analysis and study the presence of AT.

### 4.5. Targeted Proteomics in AV Samples

An array panel of 92 CVD-related proteins was used for target discovery in 40 AV tissue (N = 16 without adipocytes, N = 24 with adipocytes) lysate samples (1 µg/µL) at the Cobiomic laboratory (Córdoba, Spain; https://cobiomicbioscience.com/, accessed on 20 January 2025) using the Proximity Extension Assay (PEA) technology (Olink^®^ Target 96 Cardiovascular III panel, [Olink^®^ Bioscience, Uppsala, Sweden]). The PEA technology has been described in detail (https://www.olink.com/). The protein expression values are presented in arbitrary units (NPX values; https://olink.com/knowledge/faq?query=what%20is%20npx, accessed on 20 January 2025) in the log2 scale. Intra- and inter-assay coefficients of variations, detection limits, and biological information for each protein are reported on the manufacturer’s website (https://www.olink.com/).

### 4.6. VICs Experiments

Human AVs from 11 male donors were used for VIC isolation. For adipogenic induction, VICs were cultured in adipogenic medium: DMEM F-12 (Life Technologies, Madrid, Spain) medium supplemented with 1% FBS (Life Technologies, Madrid, Spain), 1% penicillin and streptomycin (Life Technologies, Madrid, Spain), 10 μg/mL insulin (Sigma-Merck Life Science S.L.U., Madrid, Spain), 10 ng/mL of fibroblast growth factor 2 (FGF2), 8 μg/mL biotin (Sigma-Merck Life Science S.L.U., Madrid, Spain), 1.8 μg/mL rosiglitazione (Sigma-Merck Life Science S.L.U., Madrid, Spain), 4 μg/mL D-pantothenate (Sigma-Merck Life Science S.L.U., Madrid, Spain), 400 ng/mL dexamethasone (Sigma-Merck Life Science S.L.U., Madrid, Spain), and 110 μg/mL 3-isobutyl-1-methylxanthine (Sigma-Merck Life Science S.L.U., Madrid, Spain) for 28 days.

Lastly, a co-culture assay was performed in 2 male donor’s VICs. Control cells were cultured in 6-well plates with the same media but with 1% of FBS. Adipocyte differentiation was prolonged 21 days. In parallel, same patients’ VICs were grown in control media. At day 21, co-culture of VICs and adipogenic VICs was assessed, using the nutrition media: DMEM 25 mM D-glucose (Life Technologies, Madrid, Spain) with 0.03% FBS, 8 μg/mL biotin (Sigma-Merck Life Science S.L.U., Madrid, Spain), 0.5 μg/mL insulin (Sigma-Merck Life Science S.L.U., Madrid, Spain), and 400 ng/mL dexamethasone (Sigma-Merck Life Science S.L.U., Madrid, Spain).

For the co-culture experiments, control (VIC) and differentiated aVICs and HWA conditions’ final volume was 3 mL (Figure 7). For comparable analysis, ELISA measurements were normalized per RNA concentration of each well (with the co-culture wells having almost twice the RNA concentration of the monoculture wells).

All media were changed every 2–3 days.

### 4.7. Human White Adipocyte Experiments

Human White Adipocytes (HWAs) from the *omentum* (visceral adipose tissue) of a donor (Promocell, Heidelberg, Germany) were used for the experiments. Following the manufacturer’s protocol, cells were grown with preadipocyte growth medium (Promocell), further differentiated with the adipogenic medium used for VICs for 72 h, and lastly cultured using the nutrition media described before for 21 days. The co-culture with VICs was performed at day 21 for 7 days.

### 4.8. Cell Culture Staining

VICs subjected to adipogenic differentiation from 2 men with AR were fixed with 4% formaldehyde and used for cellular staining with filtered oil-red-o solution (mixing 0.5 g/100 mL isopropanol with distilled water in a 60% and 40% proportion, respectively, Sigma-Merck Life Science S.L.U., Madrid, Spain). Microphotographs were taken of representatively stained cell fields.

### 4.9. Proteome Array Profile

Supernatants of adipogenic VICs and co-cultured VICs and adipogenic VIC were analyzed using a Proteome Profiler Human Adipokine Array Kit (ARY024, R&D Systems, Minneapolis, MN, USA). Densitometric analysis of dots was conducted utilizing Image Lab software version 5.2.1. Background subtraction was executed using the negative spots across the membrane. Following this, dot blots corresponding to duplicate specific targets were normalized to the averaged densitometry of three pairs of reference spots, adhering to the manufacturer’s instructions.

### 4.10. Enzyme-Linked Immunosorbent Assays (ELISA)

Commercial ELISA kits (R&D Systems) were used for ADAMTS1, ADAMTS5, adiponectin, aggrecan, monocyte chemoattractant protein 1 (CCL-2), CXCL-10, decorin, fatty acid-binding protein (FABP)-4, fibronectin, hyaluronan, insulin-like growth factor binding protein (IGFBP)-2, intercellular adhesion molecule 1 (ICAM-1), Interleukin (IL)-6, -12 and -32, lumican, MMP-1, MMP-2, MMP-3, MMP-7, MMP-8, MMP-9, MMP-10, MMP-13, leptin, periostin, pro Collagen I α1, resistin, syndecan-1 and -4, TIMP-1 and -2, and tumor necrosis factor (TNF)-α measurements.

### 4.11. Real-Time Reverse Transcription PCR

A Script Advanced cDNA Synthesis Kit for RT-qPCR (BioRad, Hercules, CA, USA) was used for cDNA synthesis, and iQ SYBR Green Supermix (BioRad, Hercules, CA, USA) was used for quantitative PCR assay. Therefore, we quantified the relative expression of the genes described in Supplemental Table S1.

Relative quantification of gene expression was represented by normalizing the target’s readout to the Ct readout of the housekeeping genes *RNA18S1*, *ACTB*, *GADPH*, and *HPRT*.

### 4.12. Gelatin Zymography

Aliquots of AV tissue containing 15 μg of proteins were resolved on a 10% SDS polyacrylamide gel containing 0.3% gelatin. The gel was rinsed 3 times for 15 min with a solution of 2.5% Triton X 100 to remove SDS and renature the proteins, followed by incubation for 48 h at 37 °C in 1000 mmol/L Tris-HCl, pH 7.5 with 1000 mmol/L CaCl_2_ and 5000 mmol/L NaCl to promote degradation of gelatin. Gels were fixed in 40% methanol and 10% acetic acid and then stained for 30 min in 0.25% Coomassie blue R-250 to determine the proteolytic activity of MMP-2.

### 4.13. Statistical Analysis

A normal distribution was determined by calculating Shapiro–Wilks and Kolmogorov–Smirnov tests. Data are presented as boxplots and original values within. In tissue graphs, each dot represents one donor, while for in vitro experiments, each dot represents a cell culture replicate. Normally distributed data were analyzed using the univariate two-tailed Student’s *t* test (two group comparison) or one-way analysis of variance (multiple comparisons ANOVA), as appropriate. ANOVA post-hoc analysis included Tukey or T3 Dunnet testing, as appropriate. Non-parametric tests were used for non-normally distributed data: Wilcoxon/Mann–Whitney U test or the Kruskal–Wallis tests, as appropriate. Statistical significance was accepted at *p* < 0.05. Categorical variables were represented as percentages and analyzed using either the χ^2^-test or Fisher’s exact test, as appropriate. Analyses and graph plotting were performed using GraphPad Prism 9.0 or SPSS 19.0 for Windows statistical packages.

## 5. Conclusions

AVs presenting AT exhibited changes in the inflammatory profile as well as in ECM composition and remodeling and increased thickness. Our data suggest that VICs from patients with AR had higher potential to undergo adipogenic differentiation, contributing to inflammatory ECM changes relevant to AR. Thus, VIC differentiation/recruitment into adipocyte phenotypes could be proposed as a new pathological mechanism involved in ECM disarray observed in AR. New studies unravelling the mechanistic and clinical differences among adipogenic AR and non-adipogenic AR are guaranteed.

## Figures and Tables

**Table 1:** General and echocardiographic characteristics of patients with AR according to the presence of adipose tissue in the aortic valves.

	Total	Presence of AT	Absence of AT	*p*-Value
%, (N)	100 (144)	66.67 (96)	33.33 (48)	
Baseline characteristics				
Male sex %, (N)	71.53 (103)	73.96 (71)	66.67 (32)	0.36
Age [mean (SD)]	67.07 (0.95)	67.4 (1.17)	66.35 (1.63)	0.61
Weight [mean (SD)]	75.85 (1.28)	76.19 (1.56)	75.21 (2.27)	0.72
Height [mean (SD)]	164.41 (1.87)	165.86 (2.13)	161.58 (3.66)	0.28
Body surface area [mean (SD)]	1.87 (0.02)	1.88 (0.02)	1.85 (0.03)	0.54
Hypertension %, (N)	70 (101)	72 (69)	67 (32)	0.4
Atrial fibrillation %, (N)	26 (37)	26 (25)	25 (12)	0.6
Diabetes %, (N)	12 (17)	10 (10)	15 (7)	0.49
Smokers %, (N)	32 (46)	32 (31)	31 (15)	0.84
CAD %, (N)	17 (24)	16 (15)	19 (9)	0.67
Drug medicines				
ACE/ARA %, (N)	67 (96)	64 (61)	73 (35)	0.33
Beta Blockers %, (N)	53 (76)	49 (47)	60 (29)	0.24
Statins %, (N)	44 (63)	42 (40)	48 (23)	0.54
Furosemide %, (N)	38 (55)	38 (36)	40 (19)	0.88
Biochemical analyses				
Total cholesterol (mg/dL) [mean (SD)]	172.3 (3.4)	169.2 (4.5)	178.5 (5.1)	0.21
LDL (mg/mL) [mean (SD)]	107.0 (3.0)	105.8 (3.8)	109.6 (4.7)	0.54
HDL (mg/mL) [mean (SD)]	46.73 (1.62)	46.29 (2.12)	47.61 (2.41)	0.7
Triglycerides (mg/mL) [mean (SD)]	108.2 (4.9)	103.3 (5.2)	118.7 (10.4)	0.14
Echocardiographic parameters				
LA diameter (mm) [mean (SD)]	44.18 (0.89)	44.5 (1.17)	43.57 (1.33)	0.63
LVEDD (mm) [mean (SD)]	61.52 (0.82)	61.78 (1.03)	60.98 (1.34)	0.65
LVESD (mm) [mean (SD)]	43.33 (1)	43.47 (1.28)	43.05 (1.57)	0.84
LVEDV (mL) [mean (SD)]	194.2 (9.78)	195.45 (13.67)	192.2 (13.35)	0.87
LVEF% [mean (SD)]	53.24 (1.05)	53.15 (1.34)	53.40 (1.70)	0.91
SPAP (mmHg) [mean (SD)]	37.58 (0.93)	37.39 (1.11)	38 (1.71)	0.76
Aortic dimensions (Echo)				
Annulus (mm) [mean (SD)]	26.42 (0.79)	27.07 (1.06)	24.86 (1.76)	0.2
Sinus of Valsalva (mm) [mean (SD)]	42.04 (0.82)	42.81 (1.07)	40.73 (1.23)	0.22
Sinotubular junction (mm) [mean (SD)]	40.21 (1.54)	40.43 (1.06)	39.7 (1.65)	0.71
Ascending aorta (mm) [mean (SD)]	45.69 (0.85)	45.78 (0.97)	45.53 (1.69)	0.89
Aortic dimensions (MDCT)				
Annulus (mm) [mean (SD)]	26.84 (0.39)	26.56 (0.39)	27.41 (0.88)	0.31
Sinus of Valsalva (mm) [mean (SD)]	42.87 (0.77)	43.05 (0.97)	42.48 (1.27)	0.73
Sinotubular junction (mm) [mean (SD)]	36.17 (0.87)	36.54 (1.12)	35.45 (1.36)	0.55
Ascending aorta (mm) [mean (SD)]	46.13 (1.02)	46.81 (1.25)	44.74 (1.77)	0.34
Bicuspid Valve %, (N)	16 (23)	15 (14)	19 (9)	0.68
Valve thickness (Echo/MDCT)				
Thickened Valve %, (N)	49 (70)	61 (59)	23 (11)	0.000
Valve thickness (mm) [mean (SD)]	2.97 (0.06)	3.10 (0.07)	2.67 (0.09)	0.001

ACE = angiotensin-converting enzyme inhibitors; ARA = angiotensin II receptor antagonists; AT = adipose tissue; CAD = coronary artery disease; HDL = high-density lipoprotein; LDL = low-density lipoprotein; LA = left atria; LVEDD = left ventricular end-diastolic diameter; LVEDV = left ventricular end-diastolic volume; LVEF = left ventricular ejection fraction; LVESD = left ventricular end-systolic diameter; MDCT = multi-detector computed tomography; SPAP = systolic pulmonary artery pressure.

## Data Availability

Data are available on request from the corresponding authors.

## Supplementary Materials

The supporting information can be downloaded at: https://www.mdpi.com/article/10.3390/ijms26073128/s1.
