# Supplementary material for: The Presence of Adipose Tissue in Aortic Valves Influences Inflammation and Extracellular Matrix Composition in Chronic Aortic Regurgitation
Source: Int J Mol Sci. 2025 Mar 28;26(7):3128. doi: 10.3390/ijms26073128 (PMC11989201; doi:10.3390/ijms26073128)
Supplement: Supplementary file 1 [file ijms-26-03128-s001.zip › ijms-3456490 supplementary.pdf]

Supplemental table S1: Primer sequences used for qPCR

**Target gene**	**Forward sequence**	**Reverse sequence**
RNA18S1	CGCCGCTAGAGGTGAAATTC	TCTTGGCAAATGCTTTCGC
ACTA2	ACTGCCTTGGTGTGTGACAATGG	TGGTGCCAGATCTTTTCCATG
ACTB	GCCGCCAGCTCACCAT	TCGATGGGGTACTTCAGGGT
CEBPA	GAGGAGGATGAAGCCAAGC	CAGGTGCATGGTGGTCTG
COL1A1	GGACACAGAGGTTTCAGTGGT	CACCATCATTTCCACGAGCA
COL3A1	CTTCTCTCCAGCCGAGCTTC	TGTGTTTCGTGCAACCATCC
COL4A1	CTCTGGCTGTGGCAAATGT	TTGTCCCTTTTGTTCCAGGT
FABP4	TACTGGGCCAGGAATTTGAC	TTTCCATCCCATTTCTGCAC
GAPDH	ACCAGCCCCAGCAAGAGCACAAG	TTCAAGGGGTCTACATGGCAACTG
HOXC8	GTCTCCCAGCCTCATGTTTC	TCTGATACCGGCTGTAAGTTTGT
HPRT	TTGCTTTCCTTGGTCAGGCA	ATCCAACACTTCGTGGGGTC
LPL	CAGCAAAACCTTCATGGTGA	CATTGGAGTCTGGTTCTCTCTTG
PAT2	ACTAGAAGCCAACCCCAACG	ACACACAGCAGAAGCCAAGT
PLIN1	AGGGAAGAAGTTGAAGCTTGAGG	TTCTGGAAGCATTCGCAGGT
PPARG	ACAGATCCAGTGGTTGCAGA	TGAGGCTTATTGTAGAGCTGAGTC
TCF21	CACTTCTTTCAGGTCACTCTCG	GCTACATCGCCCACTTGAG
TBX1	CCGAGATGATCGTCACCAAG	AGCATATAGTCGGCCATGG
UCP1	TCTCAGGATCGGCCTCTACG	TGCCACTCCTCCAGTCGTTA
VIM	CCTTGAACGCAAAGTGGAAT	TTGGCAGCCACACTTTCATA
